# A correlation analysis on the postpartum anxiety disorder and influencing factors in puerperae with gestational diabetes mellitus

**DOI:** 10.3389/fendo.2023.1202884

**Published:** 2023-11-27

**Authors:** Xun Zeng, Xiaofen Yan, Yan Yang, Zhangqing Peng, Shiyao Wei, Jinxia Chen, Fengchun Wu, Jiebing Chen, Ming Zhao, Chunqi Luo

**Affiliations:** ^1^ Out-Patient Department, The First Affiliated Hospital, Sun Yat sen University, Guangzhou, China; ^2^ Deparment of Private Medical Center, The First Affiliated Hospital, Sun Yat-sen University, Guangzhou, China; ^3^ Department of Internal Medicine, Zhongshan Hospital of Traditional Chinese Medicine, Zhongshan, China; ^4^ Department of Obstetrics and Gynecology, The First Affiliated Hospital, Sun Yat-sen University, Guangzhou, China; ^5^ Department of Oral Implantology, Hospital of Stomatology, Sun Yat-sen University, Guangzhou, China; ^6^ Department of Psychiatry, the Affiliated Brain Hospital of Guangzhou Medical University, Guangzhou, China; ^7^ Department of Psychiatry, Guangdong Engineering Technology Research Center for Translational Medicine of Mental Disorders, Guangzhou, China; ^8^ Out-Patient Department of Nansha Division, The First Affiliated Hospital, SunYat-sen University, Guangzhou, China

**Keywords:** gestational diabetes, postpartum, anxiety, influencing factors, correlation

## Abstract

**Objective:**

The aim of this study is to discuss the postpartum anxiety disorder and influencing factors in puerperae with gestational diabetes mellitus (GDM) to provide a clinical basis for better early identification and intervention of adverse mood.

**Methods:**

Convenient sampling method was adopted to investigate 205 pregnant women as the observation group and 201 normal healthy pregnant women in the same period as the control group. The self-rating anxiety scale (SAS) was used to investigate and observe the respondents, evaluate the postpartum anxiety status of patients with GDM, and analyze the related influencing factors. Statistical analysis of the data was performed using SAS 3.0 software. A proposed P < 0.05 was considered as statistically significant.

**Results:**

Patients with GDM had a higher risk than normal maternal anxiety, related to years of education, triglycerides, 1-h postprandial blood glucose, and a history of induced abortion.

**Conclusion:**

GDM can lead to the occurrence of postpartum anxiety, and the poor psychological state is not conducive to the maternal and infant health. Early identification and early intervention can reduce the harm caused by anxiety and promote the progress of maternal and infant health and clinical research.

## 1 Introduction

As one of the most common complications of pregnancy, gestational diabetes mellitus (GDM) (1) refers to an abnormal glucose metabolism that occurs during pregnancy, excluding the diabetes mellitus preexisting before pregnancy. GDM is associated with various factors like insulin resistance, genetics, changes in diet, and lifestyle during pregnancy (2). According to relevant literature, the prevalence rate of GDM is 14.0% globally (3). The puerperae with GDM are faced with many potential adverse pregnancy outcomes, such as macrosomia, neonatal hypoglycemia, progeny obesity, and type II diabetes, which may lead to an increased risk of mental disorders (4). According to current studies, about 10% to 15% of healthy women suffer from postpartum depression (PPD) after delivery in developed countries (5), whereas the rate was up to 24% in developing countries (6). PPD is often complicated with anxiety (7–12), which usually occurs within 6 months after delivery (13) with an incidence rate 14%–16% (14, 15). Maternal postpartum anxiety may cause some problems compromising the motor development of infant (16) as well as the breastfeeding behavior and breast milk composition (17). Therefore, for patients with GDM, who are at a high risk for mental disorders, early identification of postpartum anxiety is essential. Although current studies have proved that GDM is an important factor leading to postnatal anxiety (18, 19), most of the relevant studies merely focus on PPD, whereas postpartum anxiety and related influencing factors in puerperae with GDM are rarely reported. In this study, the relationship between GDM and postpartum anxiety was first established through a survey, and, also, relevant influencing factors were analyzed. The study results are helpful for early identification of the high-risk factors and early clinical intervention of patients with GDM with postpartum anxiety.

## 2 Materials and methods

### 2.1 Subjects

A total of 205 puerperae with GDM treated in the Obstetrics Clinic of The First Affiliated Hospital of Sun Yat-sen University between June 2021 and June 2022 were selected as the observation group, and, for statistical power, 201 healthy pregnant women in the same period were selected as the control group for postpartum anxiety investigation. Sampling method: convenience sampling. Inclusion criteria for the observation group: ① adult puerperae aged 18–49 years old and diagnosed as GDM; ② re-examined 42 days after delivery with the ability to complete the questionnaire independently; ③ without past history of systemic complications like mental disease and nervous system disease; and ④ voluntarily received and cooperated with the survey. Exclusion criteria for the observation group: ① with a history of mental illness before delivery; ② with other pregnancy complications; ③ with endocrine disease, liver or kidney dysfunction, etc.; and ④ with cognitive dysfunction. Inclusion criteria for the control group: ① healthy maternal aged 18–49 years and ② those who voluntarily received and cooperated with the survey. Exclusion criteria for the control group: ① with a history of mental illness before pregnancy; ② with other pregnancy complications; ③ with endocrine disease, liver or kidney dysfunction, etc.; and ④ with cognitive dysfunction.

### 2.2 Methods

Questionnaire of general information: A self-designed questionnaire of general information (including age, education years, monthly family income, number of children, intervention mode, and etc.) was adopted.

Clinical data: The patients were investigated for weeks of labor, gestational age, number of pregnancies, number of deliveries, history of adverse pregnancy, history of abortion, GDM, body mass index (BMI), Glycosylated hemoglobin (HBA1c), 1-h plasma glucose (1h-PG), triglyceride, and history of diabetes.

Anxiety scale: Self-rating anxiety scale (SAS) (20) formulated by Zung was used for relevant assessment. SAS consists of 20 items, for which Likert 4-grade scoring method was adopted: Scores 1 to 4 represent “never or seldom,” “a small amount of time,” “considerable time,” and “most of or all of the time,” respectively, and the total score multiplied by 1.25 was the scale standard score, which was positively correlated with anxiety. If the standard score is ≥50, then there was anxiety disorder. According to relevant literature, the reliability of this scale was 0.82 (20).

### 2.3 Statistical analysis

Excel 2003 software was used for double entry of questionnaire and SAS 3.0 for statistical analysis of data. Continuous variables were expressed as x ± s, and, Mann-Whitney U-test, a non-parametric test, was used for relevant statistical inference. Categorical variables were described by rate or percentage, and chi-square test was used for relevant analysis. The influencing factors of anxiety were discussed by binary logistic regression analysis and generalized linear mixed model, inspection level α = 0.05.

### 2.4 Ethical statement

This study was approved by the Ethics Committee of The First Affiliated Hospital of Sun Yat-sen University (approval number: Lunshen (2021)566-1), and all of the patients signed the informed consent form.

## 3 Results

### 3.1 General information of puerperae in the observation group and control group

Mann–Whitney U-test was used for continuous variables, and chi-square test was used for categorical variables (see **Table 1**). There were 205 subjects in the GDM group and 201 subjects in the healthy control group. There was statistical significance for differences in age, gestational age, 1h-PG, gestational HBA1c, gestational triglyceride, SAS, history of induced abortion, history of diabetes, and blood glucose control. For the differences in age, gestational age, 1h-PG, gestational HBA1c, gestational triglyceride, SAS, and history of induced abortion between the two groups, P = 0.020, and, for the difference in history of diabetes between the two groups, P = 0.010. The mean age, 1h-PG, gestational HBA1c, gestational triglyceride, and SAS of GDM group were higher than that of the control group, whereas the mean gestational age of GDM group was lower than that of the control group. The proportion of induced abortion history and diabetes history of GDM group was higher than that of the control group. As for BMI, education years, employment status, number of pregnancies, number of deliveries, number of children, payment method, monthly family income, and history of spontaneous abortion, there were no differences between the two groups.

**Table 1:** Comparison of basic data between the two groups.

Variables	GDM group (n = 205)	Control group (n = 201)	z	P
	Mean ± SD	Mean ± SD		
Age (years)	33.93 ± 3.86	32.42 ± 4.14	**−3.692**	**<0.001**
Gestational age (weeks)	37.79 ± 2.74	38.69 ± 1.31	**−4.503**	**<0.001**
Educational age	15.78 ± 2.62	15.98 ± 2.35	**−**0.659	0.510
1h-PG	9.19 ± 2.11	7.43 ± 1.05	**−9.188**	**<0.001**
Glycosylated hemoglobin	5.05 ± 0.47	4.83 ± 0.34	**−5.967**	**<0.001**
Triglyceride	2.32 ± 0.94	2.04 ± 1.22	**−3.673**	**<0.001**
SAS	40.05 ± 7.74	35.93 ± 7.20	**−5.205**	**<0.001**
	GDM group	Control group		P
	N (%)	N (%)		
1	59 (28.8)	38 (18.9)	**5.443**	**0.020**
2	146 (71.2)	163 (81.1)		
History of spontaneous abortion				
1	28 (13.7)	28 (13.9)	0.006	0.937
2	177 (86.3)	173 (86.1)		

GDM, gestational diabetes mellitus; 1h-PG, 1-h plasma glucose; SAS, self-rating anxiety scale; P, positive subscore.

Bold values implies statistical significance.

### 3.2 Binary logistic regression analysis on influencing factors of anxiety in puerperae

The binary logistic regression analysis was conducted with whether anxious or not as the dependent variable and general information as the independent variable, and, for categorical variables, the last was taken as the reference category. The results of univariate analysis for anxiety showed that education years, 1h-PG, and GDM were related to anxiety, with education years as a protective factor and with 1h-PG and GDM as risk factors. The Odds Ratio (OR) value for education years was 0.846 (95% CI, 0.732 to 0.977; P = 0.023), that is, with the increase of education years, the risk of anxiety decreased. For 1h-PG, the OR value was 1.227 (95% CI, 1.057 to 1.424; P = 0.007), that is, the risk of anxiety increased with 1h-PG. The OR value for GDM was 15.093 (95% CI, 3.539 to 64.373; P < 0.001), i.e., patients with GDM had an increased risk of anxiety as compared with those without GDM (see **Table 2** for details).

**Table 2:** Univariate logistic regression analysis of anxiety disorder in the two groups.

Factors	B	S.E.	Wald	Sig.	OR (95% CI)
Age (years)	0.037	0.047	0.627	0.428	1.038 (0.947, 1.137)
Gestational age (weeks)	−0.083	0.058	2.013	0.156	0.921 (0.821, 1.032)
Educational age	−0.167	0.074	5.157	**0.023**	0.846 (0.732, 0.977)
1h-PG	0.205	0.076	7.246	**0.007**	1.227 (1.057, 1.424)
Triglyceride	−0.184	0.265	0.481	0.488	0.832 (0.495, 1.399)
History of Abnormal pregnancy	0.218	0.637	0.117	0.732	1.244 (0.357, 4.337)
History of induced abortion	0.726	0.402	3.268	0.071	2.068 (0.941, 4.546)
History of spontaneous abortion	−0.814	0.747	1.186	0.276	0.443 (0.102, 1.917)
History of diabetes	0.562	0.409	1.886	0.170	1.754 (0.786, 3.914)
GDM	2.714	0.740	13.451	**<0.001**	15.093 (3.539, 64.373)

GDM, gestational diabetes mellitus; 1h-PG, 1-h plasma glucose; Sig., significance test.

Bold values implies statistical significance.

### 3.3 Multiple linear regression analysis on influencing factors of anxiety in puerperae

**Figure 1:**
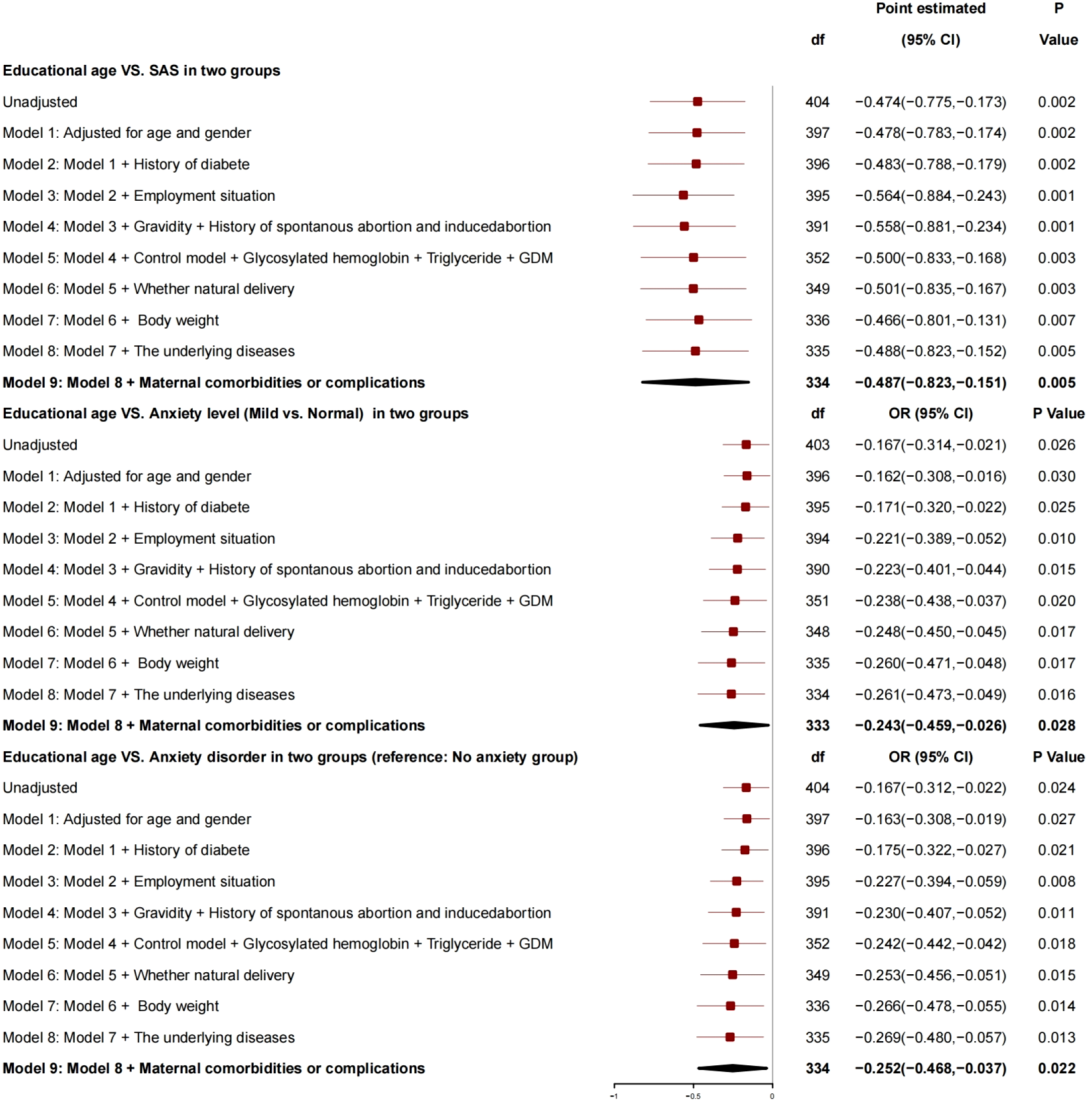
Generalized linear mixed model: Educational age and SAS.

After adjustment for age, gender, history of diabetes, employment status, number of pregnancies, history of spontaneous abortion or induced abortion, blood glucose control, HBA1c, triglyceride, GDM, normal delivery or not, and newborn weight, the generalized linear mixed model (**Figure 1**) showed that there was a correlation between educational age and SAS: For every 1 year increase, SAS decreased by 0.487 (95% CI, −0.823 to −0.151; P = 0.005), the risk of anxiety grade (mild *vs*. normal) decreased by 0.243 (95% CI, −0.459 to −0.026; P = 0.028), and the risk of anxiety disorder decreased by 0.252 (95% CI, −0.468 to −0.037; P = 0.022), and, so, it was a protective factor for anxiety.

**Figure 2:**
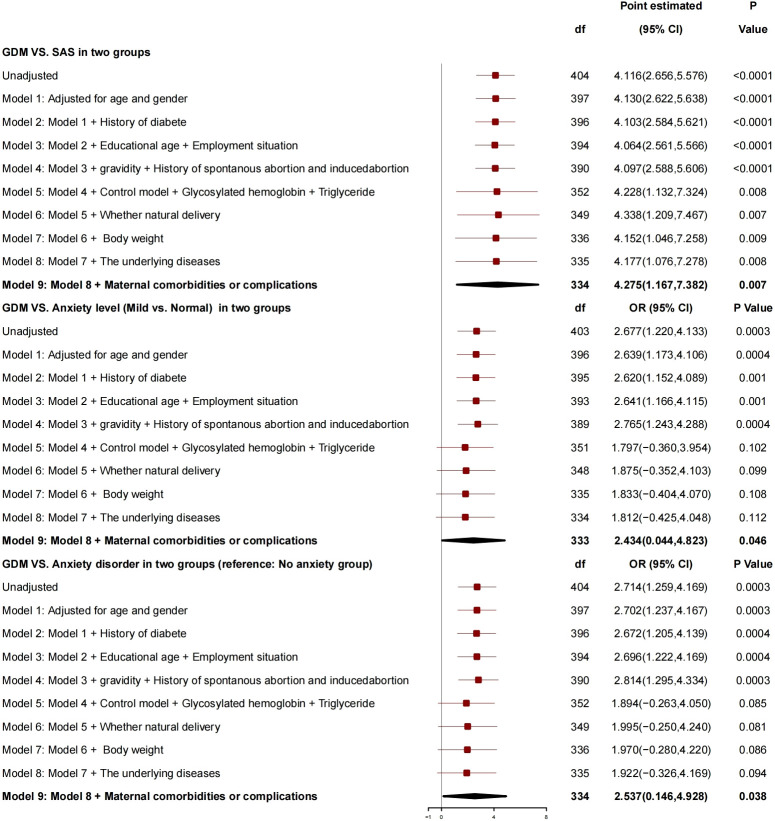
Generalized linear mixed model: GDM and SAS.

After adjustment for age, gender, history of diabetes, education years, employment status, number of pregnancies, history of spontaneous abortion or induced abortion, blood glucose controlled or not, HBA1c, triglyceride, normal delivery or not, and neonatal weight, the generalized linear mixed model (**Figure 2**) showed that GDM was correlated with SAS, and, compared with patients without GDM, patients with GDM had SAS increased by 4.275 (95% CI, 1.167 to 7.382; P = 0.007), risk of anxiety grade (mild *vs*. normal) increased by 2.434 (95% CI, 0.044 to 4.823; P = 0.046), and risk of anxiety disorder increased by 2.537 (95% CI, 0.146 to 4.928; P = 0.038), which is a risk factor for anxiety.

**Figure 3:**
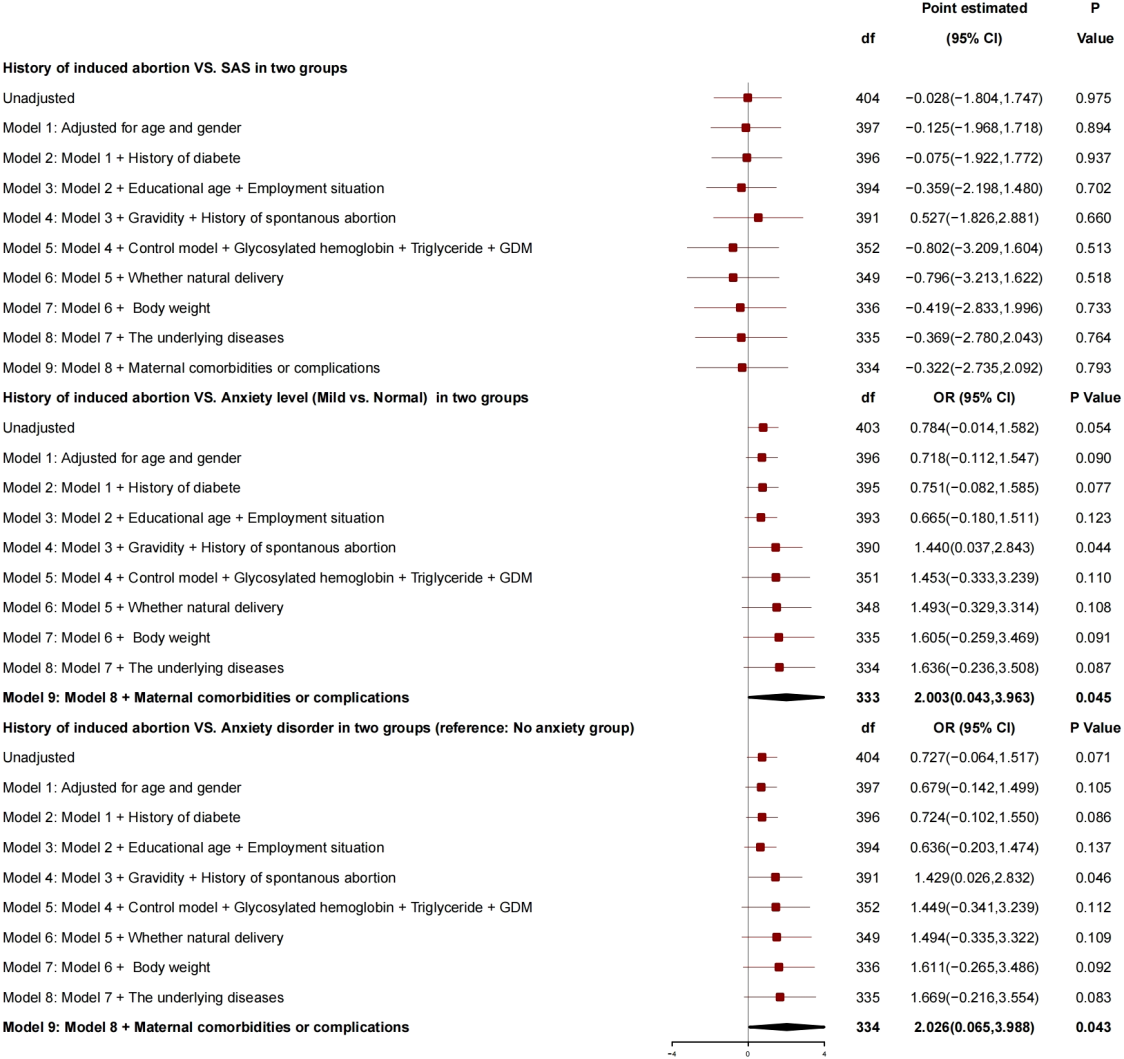
Generalized linear mixed model: History of induced abortion and SAS.

After adjustment for age, gender, history of diabetes, education years, employment status, number of pregnancies, history of spontaneous abortion, blood glucose controlled or not, HBA1c, triglyceride, GDM, normal delivery or not, and neonatal weight, there was no correlation between history of induced abortion and SAS (**Figure 3**), whereas there was a correlation between history of induced abortion and anxiety grade (mild *vs*. normal), and, compared with patients without history of induced abortion, those with history of induced abortion had risk of anxiety grade (mild *vs*. normal) increased by 2.003 (95% CI, 0.043 to 3.963; P = 0.045) and risk of anxiety disorder increased by 2.026 (95% CI, 0.065 to 3.988; P = 0.043), and, so, it was a risk factor for anxiety.

**Figure 4:**
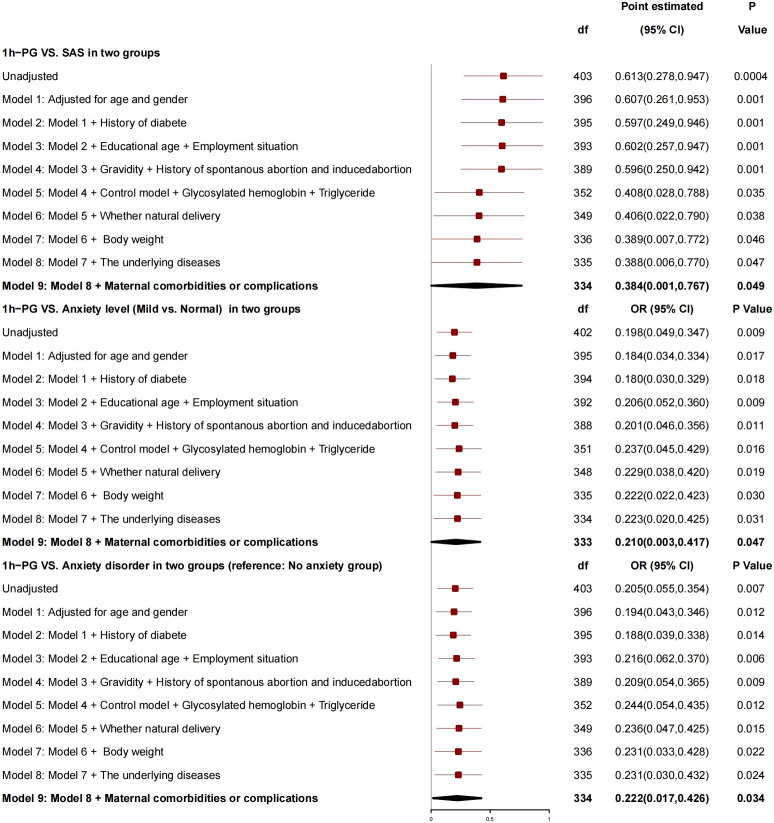
Generalized linear mixed model: 1h-PG and SAS.

After adjustment for age, gender, history of diabetes, education years, employment status, number of pregnancies, history of spontaneous abortion or induced abortion, blood glucose controlled or not, HBA1c, triglyceride, normal delivery or not, and newborn weight, 1h-PG was correlated with SAS (**Figure 4**), for every 1-unit increase in 1h-PG, SAS increased by 0.384 (95% CI, 0.001 to 0.767; P = 0.049), risk of anxiety grade (mild *vs*. normal) increased by 0.210 (95% CI, 0.003 to 0.417; P = 0.047), and risk of anxiety disorder increased by 0.222 (95% CI, 0.017 to 0.426; P = 0.034), and, so, it was a risk factor for anxiety.

**Figure 5:**
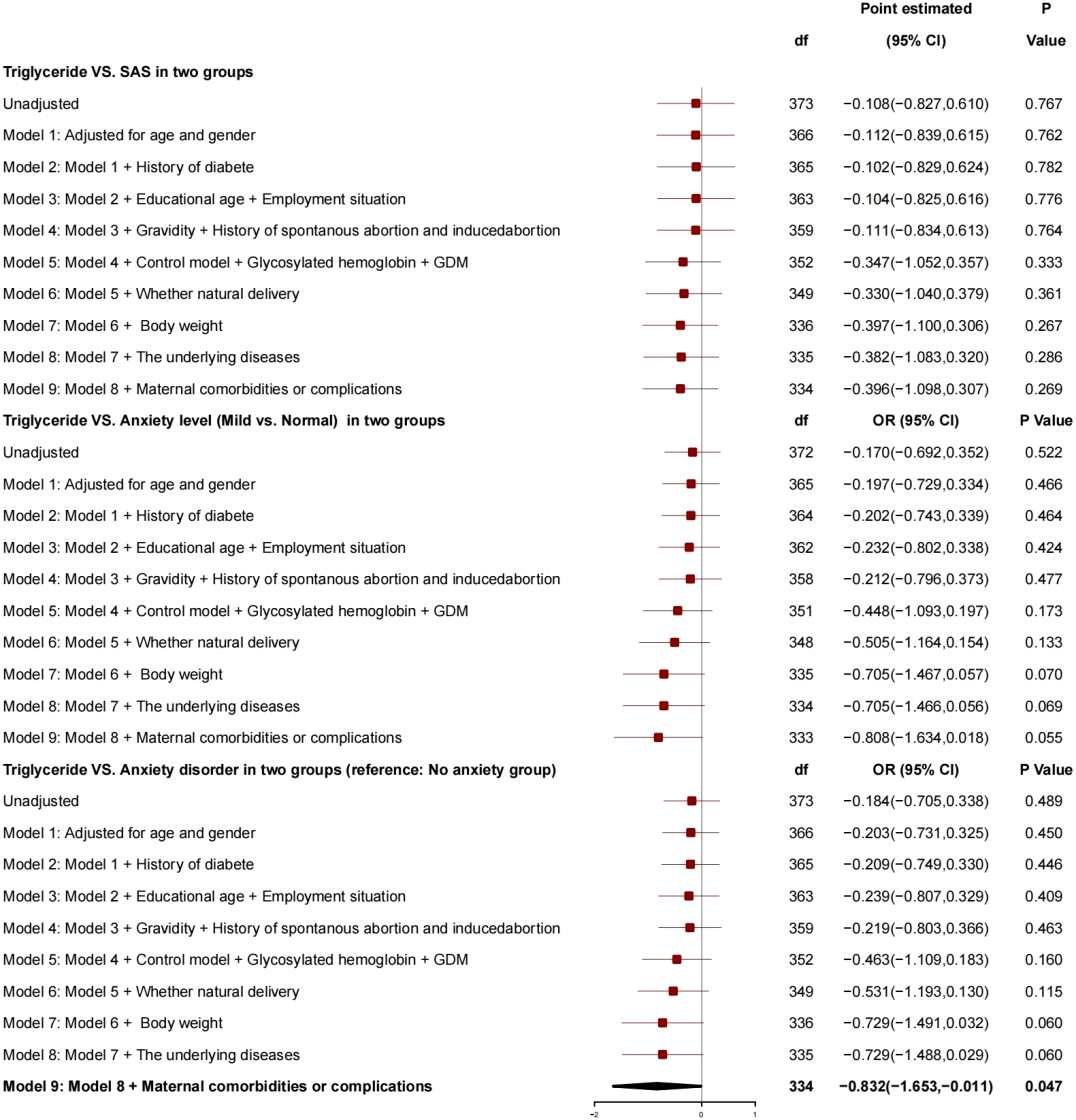
Generalized linear mixed model: Triglyceride and SAS.

After adjustment for factors like age, gender, diabetes history, education years, employment status, number of pregnancies, history of spontaneous or induced abortion, blood glucose controlled or not, HBA1c, GDM, normal delivery or not, and newborn weight, the triglyceride had no correlation with SAS (**Figure 5**), and no correlation with anxiety grade (mild *vs*. normal) but had a correlation with anxiety disorder, and, for every 1-unit increase in triglyceride, the risk of anxiety was reduced by 0.832 (95% CI, −1.653 to −0.011; P = 0.034).

## 4 Discussion

To our knowledge, this is one of the first studies to find that the postpartum anxiety score of GDM puerperae was 40.05 ± 7.74, which was much higher than that of healthy puerperae (35.93 ± 7.20). The patients’ education years and triglyceride were protective factors, whereas GDM, history of induced abortion, and 1h-PG were related to anxiety grade as the risk factors.

Education years were a protective factor for anxiety, and, for every 1-year increase in education years, SAS decreased by 0.487, the risk of anxiety grade (mild *vs*. normal) decreased by 0.243, and the risk of anxiety disorder decreased by 0.252.

Educational age was correlated with SAS as a protective factor for anxiety of puerpera (p < 0.05), which was consistent with the results reported by relevant studies from Japan and Nigeria (11, 21, 22). With many years of education and rich knowledge reserve, the patients can understand the process of pregnancy through various scientific ways and a variety of channels, learn the pressure generated during pregnancy, identify their own physical and psychological problems, deal with problems arising in life actively, and thus find a scientific solutions to their own problems (23). At the same time, for the patients, the more the education years, the higher the ability to accept GDM related knowledge, and their anxiety would be reduced with the understanding of GDM. On the contrary, the less the education years, the higher the anxiety grade and risk.

Triglyceride showed no correlation with SAS and anxiety grade, and triglyceride was related to anxiety. For every 1-unit increase in triglyceride, the risk of anxiety disorder decreased by 0.832.

Driven by the increased resistance of insulin, estrogen, progesterone, and placental prolactin, the physiological and basic levels of plasma total cholesterol and triglyceride during pregnancy were increased to guarantee sufficient energy reserves (glucose, amino acids, and lipids) as well as full development and growth of the fetus (24). For the patients in the study group, the triglyceride level was 2.32 ± 0.94, which was higher than the normal level of puerpera and was consistent with the previous study results (25–28), but still at a normal level (29). Although a high triglyceride level significantly increased the risk of GDM as a risk factor for drug-resistant subtype of GDM (30, 31), our study found that, for every 1-unit increase of triglyceride, the risk of anxiety disorder was reduced by 0.832 (95% CI, −1.653 to −0.011; P = 0.034), which was inconsistent with those in previous studies, suggesting that an appropriate increase in the blood lipid level had a protective effect on the anxiety for the patients.

GDM and 1h-PG were correlated with SAS as risk factors for anxiety. Compared with the patients without GDM, those with GDM had SAS increased by 4.275, risk of anxiety grade increased by 2.434, and risk of anxiety disorder increased by 2.537. For every 1-unit increase in 1h-PG, SAS increased by 0.384, and 1h-PG was related to anxiety grade; for every 1-unit increase in 1h-PG, the risk of anxiety grade increased by 0.210, and the risk of anxiety grade increased by 0.222.

1h-PG and GDM were the risk factors for anxiety, and patients with GDM had an increased risk of anxiety disorder as compared with those without GDM. After inclusion of blood glucose–related indicators, including HBA1c, insulin, and Oral Glucose Tolerance Test (OGTT), 1h-PG showed a positive correlation with the anxiety of patients, and the OR value for 1h-PG was 1.227 (95% CI, 1.057 to 1.424; P = 0.007), that is, the risk of anxiety increased with 1h-PG, which was consistent with that in the study of Zhao et al. (32). An analysis of the International Association of Diabetes and Pregnancy Study Groups has clarified the importance of fasting blood glucose plus 1h-PG for the diagnosis of GDM (33). The above results have indicated that 1h-PG is critical for GDM. Therefore, the rise of 1h-PG may cause anxiety in relevant patients. In clinical nursing, we should pay more attention to the health of patients with elevated 1h-PG.

History of induced abortion showed no correlation with SAS. Compared with patient without history of induced abortion, those with history of induced abortion had risk of anxiety grade increased by 2.003, and the risk of anxiety disorder increased by 2.026, and, so, the history of induced abortion was a risk factor for anxiety. This is a significant finding and also the first report on the relationship between history of induced abortion and postpartum anxiety in patients with GDM globally. Induced abortion may lead to a series of problems, including secondary infertility, ectopic pregnancy, spontaneous abortion, premature delivery, low birth weight, and pregnancy or childbirth complications. Therefore, compared with GDM puerperae without history of induced abortion, those with history of induced abortion have a higher level of postpartum anxiety (34) and a higher incidence of anxiety and depression comorbidity. Specifically, 29% of the puerperae might suffer from severe or mild depression and anxiety comorbidity. In this study, 59 patients with GDM had history of induced abortion, accounting for 28.8%, which was 18.9% higher than that of normal puerperae. In future studies, the frequency and reasons of induced abortion may be considered to further explore the relationship between induced abortion and postpartum anxiety in patients with GDM.

There are several limitations to our study that should be considered. First, the study was conducted at one hospital, and the results may not be as widespread. Second, A self-rating scale was used in this study. Although it has passed the internal consistency test, the results are still not so objective. Third, The study results are limited by sample size. In the future, a multicenter study of large sample size will be carried out to include more pregnant women in the survey, so as to obtain more reliable conclusions.

## 5 Conclusion

For the first time, this study found the status of anxiety in GDM puerperae and the related influencing factors, which are helpful for early identification and early clinical intervention of postpartum anxiety in GDM puerperae, thus reducing relevant hazards and promoting the maternal and child health as well as the progress of relevant clinical studies.

## Data availability statement

The raw data supporting the conclusions of this article will be made available by the authors, without undue reservation.

## Ethics statement

This study was approved by the Institutional Review Board (IRB) of the he First Affiliated Hospital of Sun Yat-sen University. The studies were conducted in accordance with the local legislation and institutional requirements. The participants provided their written informed consent to participate in this study. Written informed consent was obtained from the individual(s) for the publication of any potentially identifiable images or data included in this article.

## Author contributions

All authors contributed to the study design and data interpretation. FW and JieC were responsible for management and oversight of the study. YY, CL and XZ were responsible for general omnibus data analyses and were keys contributing authors to the manuscript. XY and ZP were responsible for all research interviews and clinical chart reviews associated with this study. SW and JinC provided guidance on the design of primary analyses. CL and MZ assisted with all data collection, analysis, and writing of the manuscript. All authors contributed to the article and approved the submitted version.
